# Direct electron beam writing of silver using a β-diketonate precursor: first insights

**DOI:** 10.3762/bjnano.15.90

**Published:** 2024-08-26

**Authors:** Katja Höflich, Krzysztof Maćkosz, Chinmai S Jureddy, Aleksei Tsarapkin, Ivo Utke

**Affiliations:** 1 Ferdinand-Braun-Institut (FBH), Gustav-Kirchhoff-Str. 4, 12489 Berlin, Germany; 2 Laboratory of Mechanics for Materials and Nanostructures, Empa – Swiss Federal Laboratories for Material Science and Technology, Feuerwerkerstrasse 39, CH 3602 Thun, Switzerland

**Keywords:** focused electron beam-induced deposition, precursor, silver nanostructures

## Abstract

Direct electron beam writing is a powerful tool for fabricating complex nanostructures in a single step. The electron beam locally cleaves the molecules of an adsorbed gaseous precursor to form a deposit, similar to 3D printing but without the need for a resist or development step. Here, we employ for the first time a silver β-diketonate precursor for focused electron beam-induced deposition (FEBID). The used compound (hfac)AgPMe_3_ operates at an evaporation temperature of 70–80 °C and is compatible with commercially available gas injection systems used in any standard scanning electron microscope. Growth of smooth 3D geometries could be demonstrated for tightly focused electron beams, albeit with low silver content in the deposit volume. The electron beam-induced deposition proved sensitive to the irradiation conditions, leading to varying compositions of the deposit and internal inhomogeneities such as the formation of a layered structure consisting of a pure silver layer at the interface to the substrate covered by a deposit layer with low silver content. Imaging after the deposition process revealed morphological changes such as the growth of silver particles on the surface. While these effects complicate the application for 3D printing, the unique deposit structure with a thin, compact silver film beneath the deposit body is interesting from a fundamental point of view and may offer additional opportunities for applications.

## Introduction

Direct writing with an electron beam allows for single-step and maskfree 3D printing of sophisticated nanostructures at the nanoscale [1–4]. The process relies on the electron beam-induced fragmentation of adsorbed precursor molecules on a substrate [5–9]. The precursor is typically supplied in gaseous phase. Exploiting the different complex pathways in electron-induced chemistry (such as formation of unstable intermediates and thermal assistance in adsorption and desorption) and the different process variables involved (such as spatial and temporal electron beam exposure and precursor flux), the composition and microstructure of the deposits can be tuned for obtaining desired nanocrystalline materials [10–12]. A direct writing technique is especially interesting for the fabrication of nano-optical components, where the actual geometry in combination with the material composition governs the optical response of the device [13]. However, typically a dominant carbon portion is present in the deposit due to the use of organometallic precursors [9,14], which poses practical challenges in device design and fabrication. Accordingly, various methods to improve purity during [15–18] or after deposition [18–22] were developed. For plasmonic applications, a metallic surface layer with a thickness exceeding the skin depth is sufficient to obtain the desired functionality [23–24]. Here, skin depth refers to the penetration depth of an electromagnetic field into a (non-transparent) metallic material. While pure metal deposition by direct electron beam writing was demonstrated for gold precursors with inorganic ligands [25–26], high purity comes often at the expense of a reduced shape fidelity [9]. This is also true for the recently established direct electron beam writing of silver, which demonstrated high purities of up to 74 atom % [27] but with large surface roughness and low vertical growth rates [28–30].

For silver, only few solid metalorganic compounds exist that feature sufficient vapor pressure and stability to be delivered into and used in a vacuum chamber. To date, only the class of carboxylates led to successful implementation, including both fluorinated and non-flourinated ligands [27]. The surprisingly high content of elemental silver that was found in the deposit, despite the large number of carbon atoms in the ligands, was attributed to the thermodynamically favorable release of CO_2_ upon ligand cleavage [31].

All successfully tested silver carboxylates exhibit a generally high reactivity and sensitivity upon electron beam impact, which lead to the pronounced deposition of halos. In addition, all require relatively high substrate temperatures (well above 100 °C) in order to avoid condensation. Hence, thermal effects are expected to play an important role in deposit shape evolution with the enhanced desorption rates contributing twofold: (i) The deposit purity is improved because of the fast desorption of cleaved ligands, and (ii) the volume growth rate is decreased because of short precursor residence times. In addition, surface effects, such as enhanced dissociation due to removal of ligands by chemisorption, as well as an increased mobility of the metallic clusters leading to Ostwald ripening were assumed to play an important role [9]. In order to achieve practicable vertical growth rates, new precursors are being searched for that allow for lower process temperatures.

Here, we employ the compound (hfac)AgPMe_3_ (cf. “Experimental” section) for focused electron beam-induced deposition (FEBID). (hfac)AgPMe_3_ is a white to light yellow solid, which was used before for chemical vapor deposition [32] and for growing silver nanoparticles by atomic layer deposition [33]. Like for other silver precursors, a pronounced halo and a very high sensitivity with respect to electron beam impact are observed during dissociation with the weakly focused beam of a thermal electron emitter. However, for the first time, sufficient vertical growth rates in combination with high fidelity were achieved for a tightly focused electron beam. Compared to the typically obtained granular structure of metallic nanoparticles in a carbonaceous matrix, the deposit composition and chemistry evolution are unexpected. The resulting deposit exhibits a carbon-rich body with a surface decorated with silver nanoparticles and an interfacial layer of elemental silver at the bottom, the formation mechanism of which deserves further investigation.

## Experimental

FEBID was carried out in a Hitachi S3600 tungsten filament scanning electron microscope (SEM). The precursor compound trimethylphosphine(hexafluoroacetylacetonato)silver(I), short (hfac)AgPMe_3_ (Strem Chemicals, CAS 148630-66-4), with a stoichiometry of Ag/P/F/O/C = 1:1:6:2:8 was evaporated using a fully integrated custom-built gas injection system (GIS) consisting of chemically inert steel [28]. No injection needles were used. The components of the GIS that were in direct contact with the precursors were cleaned after each use. The cleaning involved mechanical wiping using acetone-wetted tissues, followed by a sequence of sonification in acetone and ethanol, and dry-blowing with nitrogen. Depositions were carried out on silicon with native oxide. The silicon substrates were cleaned using a sequence of sonification in acetone, ethanol, and rinsed water, and dry-blowing with nitrogen. In our deposition experiments, faint deposits were visible starting at a GIS temperature of 50 °C for spots of 5 min dwell time, turning into clearly visible deposits starting from about 60 °C. From 80 °C onwards, a good deposition rate was observed. After several hours, condensation became visible, which could be avoided by heating the substrate to a temperature of 60 °C. At this substrate temperature, the spatial selectivity of the direct writing was maintained with only a very weak contribution of purely thermal dissociation (cf. Supporting Information File 1, Figure S1, for more details). All deposits shown in the following were obtained for a GIS temperature of 80 °C and a substrate temperature of 60 °C.

Depositions with a single dwell spot duration of 5, 30, and 60 min were carried out using 15 kV primary electron energy and about 0.5 nA beam current. Rectangular patterns of 10 × 10 µm^2^ were scanned in an inward spiraling beam path with a 3 nm point-to-point pitch, a dwell time of 1 μs per point, and different numbers of passes using the Xenos Patterning software.

A typical workflow involved the deposition of an automated sequence of shapes overnight, carefully avoiding unintended electron beam impact while precursor molecules were present (cf. the section on deposit evolution in Supporting Information File 1 for more details). The high-resolution images presented in the main manuscript were taken in a field-emission Hitachi S-4800 SEM. The chemical composition of the deposits was determined using energy-dispersive X-ray (EDX) spectroscopy using a Hitachi S-4800 SEM equipped with an EDAX Genesis 4000 detector and a Tescan Mira dual-beam instrument with an EDAX EDX system.

To prove for compatibility of the tested precursor with field-emission microscopes and commercial gas injection systems, further deposition experiments were performed using a dual-beam instrument Zeiss Crossbeam 340 KMAT equipped with a commercial integrated GIS from Kleindiek based on the MM3A-EM micromanipulator platform. To increase the molecule supply rate, a GIS version without valve was employed. The GIS temperature of 80 °C could be stabilized and monitored with an external temperature control unit. Because of the specific design, the crucible cap had a lower temperature. This limited the overall available time for experiments to roughly 2 h since the evaporated precursor would start to crystallize at the crucible cap. Heating of the sample was realized with a Kleindiek MHS (Micro Heating Stage), which allowed us to keep the substrate temperature constant at 60 °C throughout the whole experiment.

The microstructure of the deposits was investigated using a ThermoFischer Themis 200 G3 aberration-corrected transmission electron microscope (TEM) operating at 200 kV. Cross-sectional TEM lamellas were prepared by a standard sample preparation protocol using a Tescan Lyra3 FIB-SEM system. The TEM overview image was stitched together from two individual images using the ImageJ stitching plugin . The data is available at: https://doi.org/10.5281/zenodo.12723370.

## Results and Discussion

**Figure 1:**
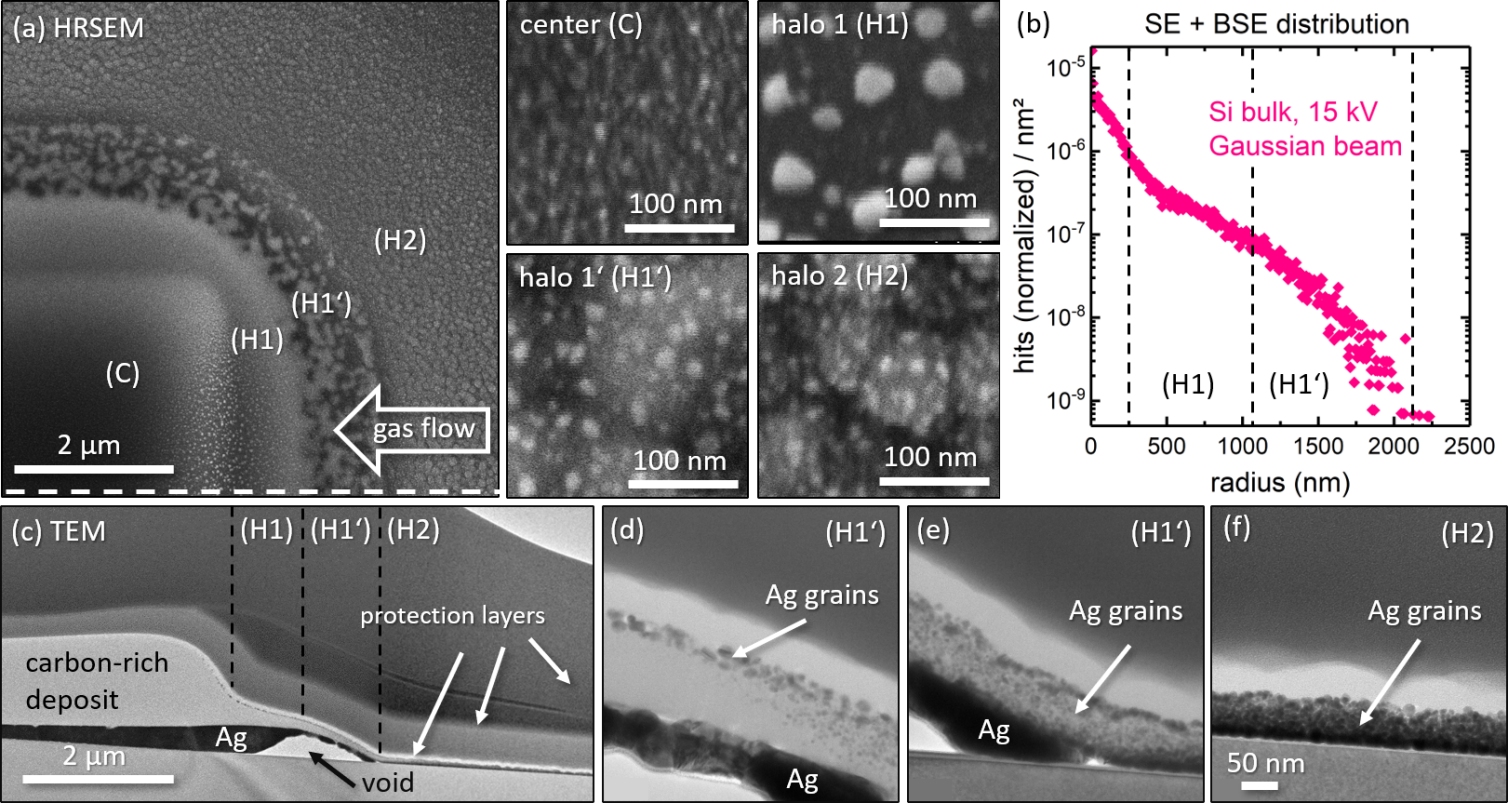
(a) Scanning electron micrograph showing the upper right quadrant of a square deposit with the different halo regions and their close-ups and (b) the corresponding simulated range of SE + BSE. The white dashed line in (a) indicates the position of the cross-sectional cut for the TEM sample preparation. (c) Transmission electron micrograph of the deposit cross-section with close-ups (d–f).

The scanning electron micrograph in Figure 1a shows one quadrant of a square deposit of 10 × 10 µm^2^ patterned in 30.000 repeats. The used beam current of 0.5 nA corresponds to an electron flux of 6 × 10^4^ electrons per second and square nanometer, and an overall dose of 1.7 μC or 1 × 10^17^ electrons per square nanometer in the center part. There is a visible halo deposition region extending several micrometers beyond the area irradiated with primary electrons, which is caused by the backscattered electrons generated by the interaction with the substrate [28]. Figure 1b shows the corresponding Monte-Carlo simulation of the secondary and backscattered electron (SE + BSE) distribution for a Gaussian beam of 250 nm FWHM impinging on a flat silicon substrate matching our deposition conditions. The density of SE and BSE decreases exponentially with increasing distance to the central beam and extends to roughly 2 µm, which corresponds to the halo region 1 (H1 and H1’) observed in Figure 1a. The fact that halo region 2 extends beyond 2 µm could be the result of two processes, namely, (i) forward scattering at the deposition edge of 1 µm height, providing an extremely small but still non-zero electron flux for dissociation, and (ii) the diffusion of incompletely dissociated precursor molecules out of halo region 1, which are only later fully decomposed.

The close-up of the central region of the deposit (C) in Figure 1 shows a dark appearance with uniformly sized nanoparticles of about 5–10 nm. When moving away from the central area, three very distinct regions regarding the halo morphology are found. The first halo region (H1) has a relatively bright appearance with particles of about 10–40 nm size that are most pronounced in the direction of the molecule delivery (indicated by the white arrow in Figure 1a). Below, smaller particles of about 5–10 nm can be seen. Particles of about 5–20 nm size are observed in the second part of the first halo region (H1’), where the background forms irregularly shaped dark and bright regions of several hundreds of nanometers in size. After taking the high-resolution images, the imaged regions showed an increase in particle size and brightness (cf. Supporting Information File 1, Figure S5j, and the corresponding explanations). This points to incomplete dissociation in the deposition process, which embeds ligand elements in the deposited material in addition to silver. Upon electron-irradiation, such material can be further dissociated, and the contained silver can form larger cluster by enhanced diffusion. Finally, the second halo region (H2) depicts similar particle sizes around below 20 nm sitting on top of larger bright areas of 50–250 nm. The composition of the different halo regions was studied by EDX. Because of the rich morphology observed in the deposit, no thin-film correction was applied. Instead, the EDX spectra were taken at different acceleration voltages to obtain independent data sets (see Supporting Information File 1 for an overview of all quantification results). The observed trend in composition was from a carbon-rich deposit in the central region to a strongly increasing silver content in the halo. This is very similar to observations for carboxylate silver precursors [27–30], where the reduced electron flux in the halo region together with the substantial substrate temperature (above 100 °C) allowed for efficient desorption of the cleaved ligands instead of their further dissociation and co-deposition into the deposit.

To further investigate the microstructure of the deposit, a thin lamella along the dashed white line in Figure 1a was prepared and studied by TEM. The TEM overview image is depicted in Figure 1c, its alignment and magnification were adapted to match the high-resolution SEM (HRSEM) image above. The deposit structure turned out to be extremely non-uniform with a continuous layer of elemental silver at the interface between deposit and silicon substrate (cf. Supporting Information File 1, Figure S4, for more details on the elemental analysis). In addition, small elemental silver grains decorating the surface of the carbon-rich deposit were found. The two very different contrast regions of the first halo regions H1 and H1’ turn out to contain a large void of about 1 μm lateral extension. Being observable for several deposits, this void is a strong indicator of gas formation during the deposition process. The halo region H1 is composed of three layers, that is, the interfacial silver layer, the carbonaceous layer, and a layer of silver particles at the top. As depicted in Figure 1d, the upper two layers merge along H1’, transitioning into the typical granular deposit structure of elemental metal particles embedded in a carbonaceous matrix sitting on top of the relatively uniform interfacial layer of elemental silver (cf. Figure 1e). The second halo region H2 depicted in Figure 1f becomes thinner overall and shows a high density of silver particles with a transition to continuous silver towards the bottom.

Similar non-uniform deposit structures were observed earlier. For pillar deposition of gold using Me_2_Au(acac) in a water atmosphere at about 1 Pa pressure, a solid metallic core surrounded by a carbon-rich shell was obtained [34]. For planar deposits, similar microstructures were obtained during platinum deposition using Pt(η^5^-CpMe)Me_3_ [35] and ruthenium deposition using (EtCp)_2_Ru [36], both in combination with post-deposition purification employing electron beam irradiation in a water atmosphere. For the case of silver deposition with a focused electron beam, such non-uniform deposit structures have not been observed before.

The mechanism of the interfacial silver layer formation in our case may include several factors. One factor may be the presence of water. In the typically used high-vacuum range of 10^−4^ Pa in scanning electron microscopes during deposition, a double layer of water is still present on hydrophilic surfaces. This turns water into a natural co-reactant of direct electron beam writing [7] and helps in the formation of neutral volatile reaction products from the ligands, such as CO, CO_2_, CH_4_ or Hhfac, removing most of the ligand elements. A second important factor here could be the thermal energy input from the elevated stage temperature of 60 °C, which increases the mobility of the formed silver atoms and clusters in the carbonaceous matrix. Finally, collisional momentum transfer from the primary electrons may enhance diffusion of silver and related reordering processes in the carbonaceous matrix [14]. Under our conditions, this energy input is about 0.3 eV for a silver atom and 2.6 eV for a carbon atom, which would be available for rearrangement mechanisms.

The clarification of the formation mechanism of the interfacial silver layer deserves further in-depth studies, which are beyond the scope of this article. These studies would involve surface science approaches using mass spectrometry and/or other spectroscopic techniques, such as X-ray photoelectron spectroscopy, and Raman or FTIR spectroscopy [5,8,37], to study the nature of the desorbed and incorporated molecular fragments ideally during the irradiation process.

Up to now, only silver pentafluoropriopionate allowed for three-dimensional growth [30]. However, the use of this compound required relatively high stage temperatures of about 155 °C (corresponding to 125 °C on the sample surface) to avoid condensation. On the one hand, temperature decreases the molecule residence times exponentially according to the Arrhenius law resulting in lower vertical growth rates [14]. On the other hand, a distinct thermal reaction contribution is expected to occur [9]. For (hfac)AgPMe_3_, stage temperatures between 55 and 60 °C were safely suppressing condensation, which improved the adsorbate residence time and, in turn, the vertical growth rates.

**Figure 2:**
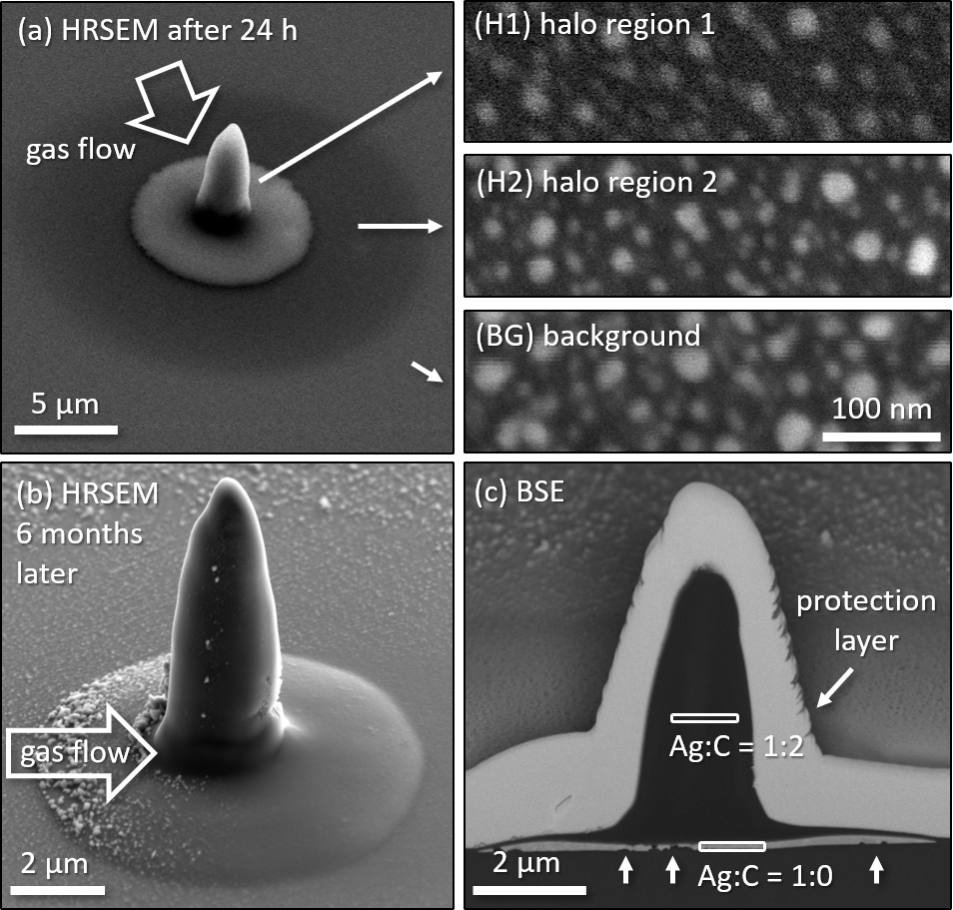
Scanning electron micrographs of a spot deposit with 60 min continuous spot irradiation (a) with the corresponding close-ups of the halo regions. (b) High-resolution SEM image 6 months later, showing grown silver crystals in the direction of the precursor gas flow and in the further surrounding. (c) BSE image from the cross section where heavy elements appear bright. The Ag/C ratios were obtained from EDX measurements at the cross section in the areas indicated by white rectangles.

Figure 2a–c shows high-resolution scanning electron micrographs of a 60 min spot deposit, where the different flux regions are indicated. The high electron flux of about 6 × 10^4^ electrons·s^−1^·nm^−2^ leads to carbon rich 3D tip growth with a volume growth rate of 0.194 µm^3^/min and a vertical growth rate of about 80 nm/min. With continuously decreasing electron flux, three different areas in the halo can be distinguished in Figure 2a. Here, halo region 1 extends significantly beyond the simulated range of SE and BSE, which can be attributed to forward scattering in the apex of the deposit [38].

EDX measurements in top view proved an efficient release of phosphorus and fluorine in the halo region but showed a significant amount of both in the carbon-rich deposit (cf. Supporting Information File 1 for more details). Of note is that the surrounding of the deposit as well as the surface topography changed after deposition. Tiny particles of few nanometers in size appeared significantly larger after 6 months, when the cross section was prepared. This, again, hints toward incompletely dissociated precursor, which may have been further dissociated after the actual deposition process (cf. the section on deposit evolution in Supporting Information File 1 for more details). The cross-sectional cut unveils a microstructure similar to that observed for the planar case, that is, a carbon-rich deposit sits on top of an elemental interfacial silver layer. Again, a tendency to form voids beneath the silver is visible, as indicated by the vertical white arrows in Figure 2c. As in the case of planar deposition, the silver thickness increases from the center towards the halo region. However, for the spot exposure, the pure silver layer extends significantly beyond the range of secondary and backscattered electrons. Hence, the silver atoms and clusters must have received sufficient kinetic energy for migration. The difference between planar and spot deposit is the thickness of the deposit itself. While the silicon substrate suppresses beam-induced heating because of its high thermal conductivity, the deposit itself is most probably a bad heat conductor [39]. Consequently, a temperature gradient with the highest temperature at the deposit surface is likely to occur [40]. This locally increased temperature mobilizes the different species and may trigger additional thermally driven chemical processes. As a result, the mobilized silver atoms/clusters would come to rest in the regions with the lowest local temperature at the sample surface.

In a next step, the usability of (hfac)AgPMe_3_ with a commercial gas injection system and a field-emission gun was assessed. In contrast to the used thermal emitter, this allows for electron beam spot sizes in the single-digit nanometer range and an extremely increased local electron flux. Given sufficient local precursor flux, this should lead to fast vertical growth that minimizes both parasitic deposition in the halo region and the unwanted contribution of the rather slow purely thermal dissociation.

Spot deposits were performed at 15 kV with 1 µs dwell time and a beam current of 246 pA. With 8 × 10^7^ electrons·s^−1^·nm^−2^ (assuming 5 nm FWHM of the beam), the local electron flux in this case was about three orders of magnitude larger than that of the thermal emitter. The resulting pillar height was more than 2 µm. Given the deposition time of 620 s (dose 1.5 × 10^−7^ C) and the pillar width of 320 nm, this corresponds to a volume growth rate of approximately 0.012 μm^3^ per minute and a vertical growth rate of about 200 nm per minute.

**Figure 3:**
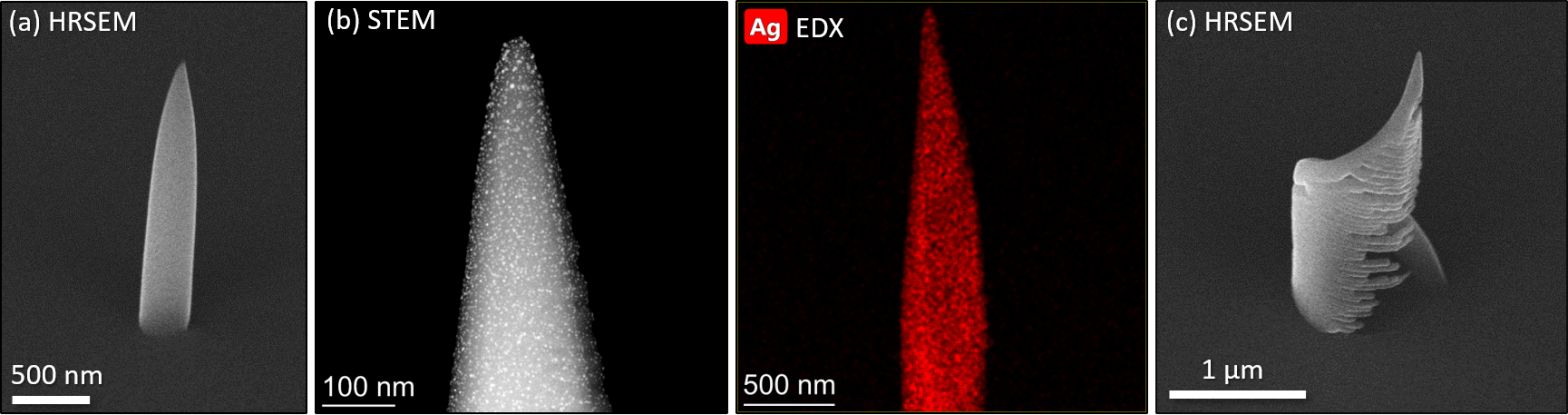
Electron micrographs of deposition results using (hfac)AgPMe_3_ in a commercial gas injection system and a field-emission microscope. (a) Pillar with smooth side walls and without noticeable halo, (b) dark-field STEM image of the pillar with silver nanocrystals appearing as bright spots with corresponding EDX map, (c) helix with a radius of 500 nm and strong parasitic co-deposition below the helix arm.

The obtained vertical growth rate was sufficient to avoid any signs of halo evolution around the structure (Figure 3a). As this growth rate was only about two times larger than that of the thermal emitter, a precursor-limited growth regime and, thus, enhanced co-dissociation of the ligands is expected.

For later TEM investigations, the deposition was carried out directly on a TEM grid. The STEM image in Figure 3 shows that a significant portion of nanoparticles sits on the deposit surface. This is in contrast to the typical deposition morphology with metal nanoparticles in a carbonaceous matrix from FEBID with organometallic precursor compounds. The obtained silver content of about 0.6 atom % is very low. Apart from the large carbon portion of about 70 atom %, remarkably high contents of oxygen (22 atom %) and phosphorous (7 atom %) are found. This is in line with the favored co-deposition of ligand elements at high local electron flux. Furthermore, compared to the initial precursor stoichiometry of Ag/P = 1:1, this implies again a strong mobility of silver, which most probably migrates from the pillar volume and enriches as an interfacial layer at the bottom.

Finally, a helix with a radius of 500 nm and one turn was deposited by scanning the electron beam in a circular path with a pitch of 10^−4^ nm and a dwell time of 1 μs. These parameters resulted in an overall deposition time of 21 min 17 s and in a beam velocity of approximately 0.15 μm per minute to match the observed vertical growth rate. The typical result is shown in Figure 3c. As a consequence of the large electron sensitivity, a significant parasitic co-deposition occurred below the actual helix wires caused by the residual primary electrons that penetrate the helix arms [39]. This can potentially be reduced by lowering the primary beam energy and, correspondingly, the interaction volume, while at the same time a more circular cross section of the wires can be achieved.

## Conclusion

In conclusion, we studied the compound (hfac)AgPMe_3_ as a novel precursor for focused electron beam-induced deposition. In contrast to the currently available carboxylate precursors, (hfac)AgPMe_3_ is the first silver precursor that is compatible with commercial gas injection systems as it operates at a temperature of 80 °C, comparable to the ubiquituous Pt precursor Pt(η^5^-CpMe)Me_3_. At a required stage temperature of about 60 °C, the residence time of the molecules is long enough to realize sizable vertical growth rates and halo-free deposition for tightly focused electron beams.While this is a promising step towards 3D printing, the ability of (hfac)AgPMe_3_ to produce smooth vertical structures comes at the cost of the silver content. In addition, (hfac)AgPMe_3_ reacts extremely sensitively to the impact of electrons, resulting in strong parasitic co-deposition below overhanging 3D wire structures. Concerning the electron-induced chemistry, a strong dependence on the electron flux is revealed, leading to sharply distinguishable chemical regimes in the halo and in the deposit body. Here, a tendency towards incomplete dissociation at low electron fluxes and co-dissociation and deposition of the ligands at high fluxes was observed. Together with a high mobility of the silver in the shape-forming carbonaceous matrix and possible further reaction pathways, this leads to the formation of a pure silver layer at the interface to the substrate. Although such a rich chemical evolution and the extreme sensitivity of the compound to electron impact is exciting from a fundamental point of view, it impedes the practical implementation for 3D FEBID. One option may include the high electron flux deposition of the 3D scaffold using (hfac)AgPMe_3_ followed by low-flux irradiation to add a layer of silver crystals using the same precursor. Finally, future research needs to identify other promising precursor candidates, ideally of the carboxylate type with long aliphatic side chains [31], to combine both stable chemistry leading to high silver contents and usability at low evaporation temperatures for 3D printing.

## Supporting Information

File 1 Additional experimental data.

## Data Availability

Additional data is available at https://doi.org/10.5281/zenodo.12723370.
