# Supplementary material for: Direct electron beam writing of silver using a β-diketonate precursor: first insights
Source: Beilstein J Nanotechnol. 2024 Aug 26;15:1117–24. doi: 10.3762/bjnano.15.90 (PMC11368048; doi:10.3762/bjnano.15.90)
Supplement: File 1 — Additional experimental data. [file Beilstein_J_Nanotechnol-15-1117-s001.pdf]

BEILSTEIN JOURNAL OF NANOTECHNOLOGY

Supporting Information

for

Direct electron beam writing of silver using a β-diketonate precursor: first insights

Katja Höflich, Krzysztof Maćkosz, Chinmai S. Jureddy, Aleksei Tsarapkin and Ivo Utke

Beilstein J. Nanotechnol. **2024,** *15,* 1117–1124. doi:10.3762/bjnano.15.90

Additional experimental data

License and Terms: This is a supporting information file under the terms of the Creative Commons Attribution License (https://creativecommons.org/licenses/by/4.0). Please note that the reuse, redistribution and reproduction in particular requires that the author(s) and source are credited and that individual graphics may be subject to special legal provisions.
The license is subject to the *Beilstein Journal of Nanotechnology* terms and conditions: (https://www.beilstein-journals.org/bjnano/terms)

The data is available at https://doi.org/10.5281/zenodo.12723370

Control experiment

To assess the influence of purely thermal reaction channels the vicinity around the actual deposition sites was also studied using high-resolution scanning electron microscope (HRSEM) imaging. The rectangular patterns and spot deposits are carried out in one automated sequence on an area that was not irradiated with electrons prior to the experiment. The surrounding region around the patterns experiences the same molecular flux but no electron exposure during the entire deposition experiment (typically over night exposure). This means, the control experiment for purely thermal decomposition is automatically included in every deposition run.

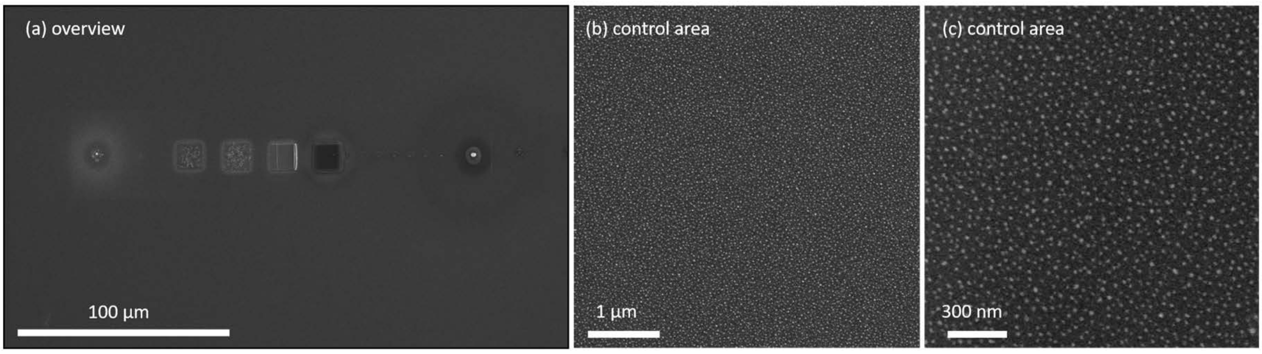

Figure S1: HRSEM images of (a) the entire patterning region and (b),(c) closeup images of the area around the patterns which serves as a control to verify the contribution of purely thermal dissociation.

Figure S1 shows a sequence of HRSEM images taken in the Hitachi S-4800 microscope after the experiment. In the overview image of Figure S1a most of the automated deposition sequence is visible. The bright spherical region on the left is the area where a first test spot deposit was done and used for appropriate focusing. Next to it the deposition sequence of the four square-shaped deposits with varying number of repeats (100, 200, 1000, and 30000) is found, followed by a sequence of 5 min spot deposits, a 60 min spot deposit, and another sequence of 5 min spots (not visible in the overview). The 5 min spots were used as a reference to ensure that the precursor was not fully consumed before the end of the experiment. Figure S1b,c displays close-up images of the area around the pattern that exhibit tiny silver particles of about 5–20 nm in size. This resembles the behaviour of the carboxylate precursors which also show a very slow contribution of chemical vapor deposition due to the elevated stage temperature. Still, the overall amount of silver is too low to detect it by energy-dispersive X-ray (EDX) spectroscopy. In addition, such become visible only after several hours of continuous gas flow (in this case more than 10 h) at an elevated stage temperature. The spatial selectivity of direct electron beam writing with this precursor is therefore not limited in practice.

Detailed EDX results

A detailed overview of all elemental analyses using energy-dispersive X-ray (EDX) spectroscopy is provided below.

Elemental analysis in high-resolution SEM

For both types of deposits, rectangular and spot deposits, all indicated regions (central part and halo regions) were characterized by EDX. All of these EDX measurements were conducted in top view in the Hitachi S-4800 electron microscope, that was used for high-resolution imaging. Acquisition of the spectra was carried out at different acceleration voltages to obtain independent data sets. The acquisition time of all spectra was 120 s with background spectra taken at a large field of view of about 100 µm while the actual deposit and halo spectra had a 250 nm field of view (max. magnification of 500k). Since no thin-film correction could be applied and since the relative error of very small element quantities becomes very large, these results have to be interpreted with great caution. Figure S2 shows the quantification results for the case of the square deposit obtained for two different acceleration voltages used for EDX where the obtained variations become immediately apparent. Still, we decided to add some data and discussion here, as the varying element ratios with electron dose may give useful hints towards further experiments to elucidate the actual reaction channels.

Atomic percentage								Composition normalized to Ag						
**10 kV**	Ag	C	O	P	F	Si		**10 kV**	Ag	C	O	P	F	Si
Center	5.3	59.6	22.5	7.6	1.9	3.1		Center	1.0	11.3	4.3	1.4	0.4	
Halo (H1)	20.9	48.1	22.6	2.8	3.4	2.3		Halo (H1)	1.0	2.3	1.1	0.1	0.2	
Halo (H1')	6.7	40.0	6.2	0.6	1.8	44.7		Halo (H1')	1.0	2.3	1.1	0.1	0.2	
Halo (H2)	3.5	24.8	2.4	0.2	1.2	67.8		Halo (H2)	1.0	7.1	0.7	0.1	0.3	
**Atomic percentage**								**Composition normalized to Ag**						
**7 kV**	Ag	C	O	P	F	Si		**7 kV**	Ag	C	O	P	F	Si
Center	0.7	64.9	24.8	8.3	0.9	0.3		Center	1.0	88.9	34.0	11.4	1.2	
Halo (H1)	21.4	47.5	24.3	3.0	1.5	2.3		Halo (H1)	1.0	2.2	1.1	0.1	0.1	
Halo (H1')	13.4	41.0	12.5	1.9	1.0	30.3		Halo (H1')	1.0	3.1	0.9	0.1	0.1	
Halo (H2)	7.3	28.0	5.3	0.7	0.7	58.0		Halo (H2)	1.0	3.8	0.7	0.1	0.1	

Figure S2: Overview of EDX quantification results obtained for the square deposit from Figure 1 in the main manuscript. In the center of the deposit the silver content is largely underestimated. We thus refer to the STEM results and the compact interfacial silver layer there. Still, the data gives a tendency to compositional changes of the carbonaceous matrix depending on the electron flux.

For the rectangular deposit depicted in Figure 1 the central deposit region (C) turned out to be carbon rich with with a large amount of oxygen, stable phosphorus content but strongly reduced fluorine compared to the initial precursor stoichiometry (Ag/P/F/O/C = 1:1.4:0.4:4.3:11.3 without background/layer correction). This corresponds to the layered structure with the elemental silver at the bottom and the carbonaceous deposit on top. In halo region H1 the silver content is strongly increased in combination with an efficient removal of phosphorus, oxygen and carbon (Ag/P/F/O/C = 1:0.14:0.17:1.1:2.36 without background/layer correction). In halo region H1' apart from a substantial silver amount again more carbon is found (Ag/P/F/O/C = 1:0.08:0.26:0.9:5.9 without back-

ground/layer correction). In halo region H2 the oxygen content is further reduced while the carbon content is increased (Ag/P/F/O/C = 1:0.07:0.34:0.7:7.1 without background/layer correction).

Atomic percentage								Composition normalized to Ag						
**10 kV**	Ag	C	O	P	F	Si		**10 kV**	Ag	C	O	P	F	Si
Center	1.5	64.8	19.7	5.4	1.9	6.7		Center	1.0	42.3	12.9	3.5	1.3	
Halo 1	26.6	40.5	15.8	2.4	2.9	11.8		Halo 1	1.0	1.5	0.6	0.1	0.1	
Halo 2	0.7	28.0	2.0	0.2	0.9	68.2		Halo 2	1.0	40.5	2.9	0.3	1.3	
Background	1.6	14.8	1.5	0.0	0.8	81.3		Background	1.0	9.0	0.9	0.0	0.5	
**Atomic percentage**								**Composition normalized to Ag**						
**7 kV**	Ag	C	O	P	F	Si		**7 kV**	Ag	C	O	P	F	Si
Center	3.2	58.0	27.6	4.8	0.6	5.8		Center	1.0	18.4	8.7	1.5	0.2	
Halo 1	27.6	42.8	22.8	3.1	2.1	1.6		Halo 1	1.0	1.6	0.8	0.1	0.1	
Halo 2	2.3	37.0	5.6	1.1	0.8	53.2		Halo 2	1.0	16.5	2.5	0.5	0.4	
Background	3.9	17.0	2.3	0.3	0.9	75.6		Background	1.0	4.4	0.6	0.1	0.2	

Figure S3: Overview of EDX quantification results obtained for the 60 min spot deposit from Figure 2 in the main manuscript. In the center of the deposit the silver content is largely underestimated. We thus refer to the HRSEM images and EDX quantification of the cross section and the compact interfacial silver layer there. Still, the data gives a tendency to compositional changes of the carbonaceous matrix depending on the electron flux.

For the spot deposit depicted in Figure 2 of the main manuscript even less silver is found in the central part as can be seen in Figure S3. The carbon-rich deposit again released most of the fluorine (Ag/P/F/O/C = 1:4:1:9-13:34-43 without background correction). The fact that per one atom of phosphorus only one fluorine atom but 2 oxygen and 8–10 carbon atoms are found (stoichiometry 1:6:2:8 in the molecule), that is, all oxygen and carbon of the ligands but mostly no fluorine, hints towards specific reaction channels during the co-deposition of the hfac ligand that deserve further investigation.
When moving to a lower electron flux regime (halo region 1), the phosphorus and carbon were efficiently released during deposition as well, resulting in high silver contents of about 30 atom % (Ag/P/F/O/C = 1:0.1:0.1:0.5:1.5 without background correction). For further decreasing electron flux (halo region 2) the carbon and phosphorus and fluorine content increased again leading to an approximate composition of Ag/P/F/O/C = 1:0.3:1.3:3:40 without background correction (attention: large relative error here due to small absolute values). The third region is close to the background with no detectable traces of phosphorus and a composition of (Ag/P/F/O/C = 1:0:0.47:1:9 without background correction). The common trend here is a certain low-electron flux regime that allows for efficient removal of most of the ligands.

Elemental analysis for the cross-sectional lamella of the square deposit

The cross section of the rectangular deposit was studied using diffraction and EDX in the transmission electron microscope (TEM). Figure S4 shows the resulting EDX maps for all precursor elements and silicon as the substrate material. The overview of the lamella is depicted in the TEM image on the upper left. The black square indicates the region from which the high-angle annular dark-field (HAADF) scanning transmission electron microscope (STEM) image and the corresponding EDX

elemental maps are taken. The red circle indicates the region from which the diffraction pattern was taken that confirms the presence of crystalline elemental silver. This central layer of elemental silver with the carbonaceous deposit on top is also visible in the maps for silver and carbon. While fluorine is detectable only in traces, still a substantial phosphorous content is visible. Oxygen is present in the native oxide layer covering the silicon substrate and in the carbonaceous deposit on top of the silver. Spurious silicon and carbon signals are found also in other regions due to multi-scattering events.

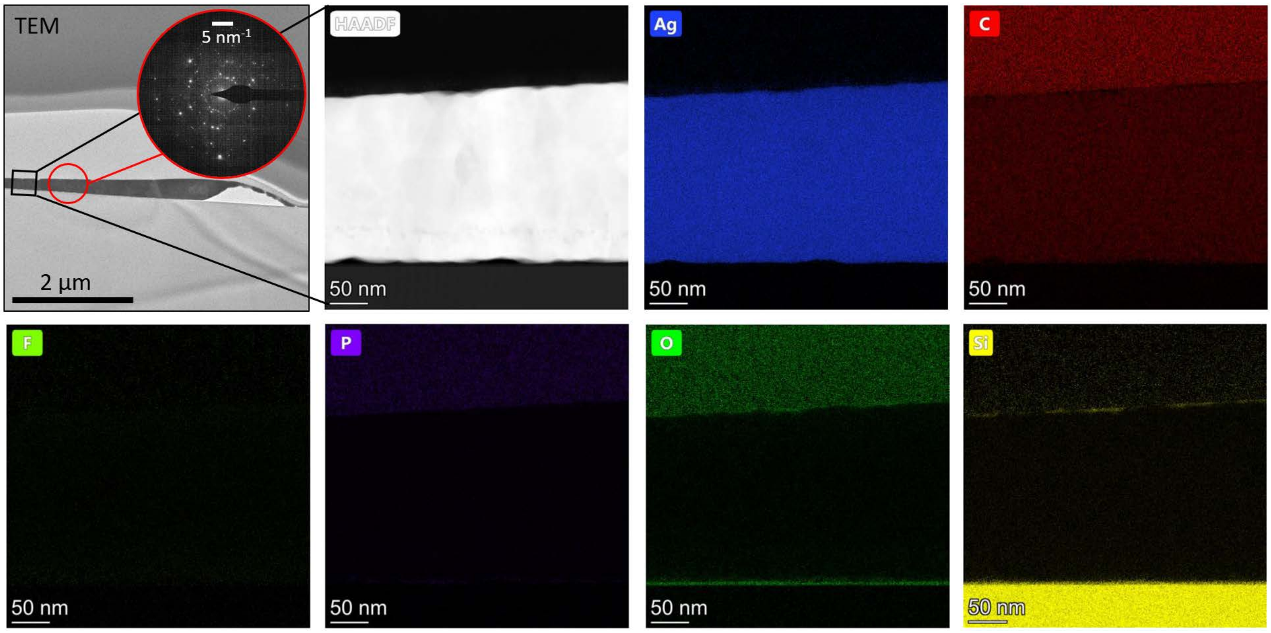

Figure S4: TEM overview image of the cross section of the rectangular deposit depicted in Figure 1 of the main manuscript with the black square indicating the region of the HAADF STEM image and corresponding EDX maps for all elements in the precursor compound and silicon. The diffraction image shown as inset in the TEM image was taken in the region indicated by the red circle.

Deposit evolution

The following sequence of images is intended to show the high sensitivity of the precursor to electron beam impact and the rich morphology evolution obtained after growth.
All low-resolution images were taken in-situ in the deposition SEM with a tungsten cathode. The high-resolution images were taken in a field-emission SEM. As the precursor is extremely sensitive to electron beam irradiation any unintended irradiation was avoided. This was realized by automated patterning of a sequence of deposits which were only imaged hours after the GIS and sample had been cooled down and the base pressure was recovered. Figures S5a,b depicts such a deposition sequence of four squares with increasing number of repeats (100, 200, 1000, and 30000) followed by a series of 5 min dots to assess a potential drop of the precursor flow over the several hours of deposition time. The feature on the very left side was deposited for focusing.

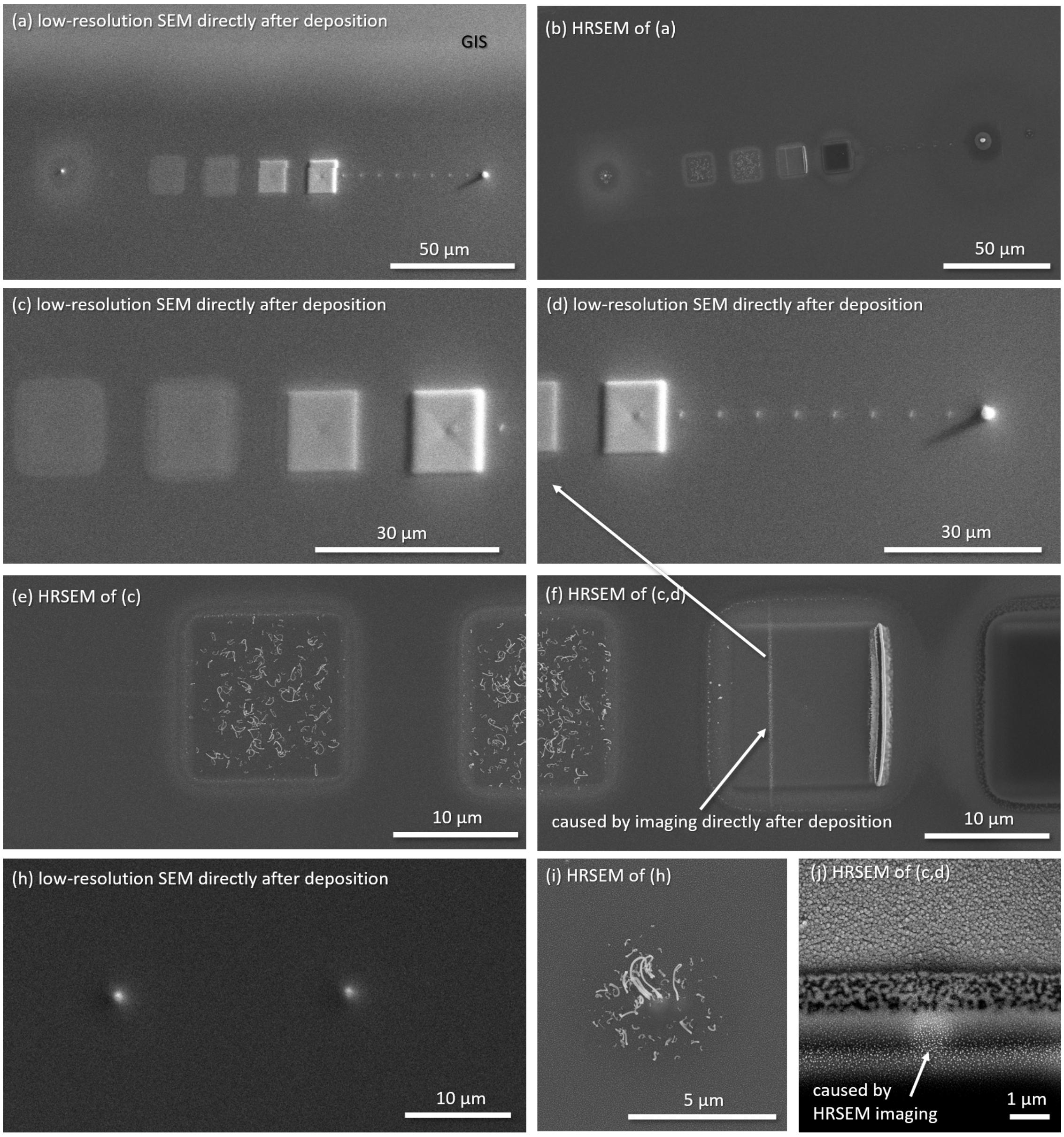

Figure S5: Scanning electron micrographs of deposits directly taken after deposition in the tungsten emitter low-resolution system compared images taken with high resolution in a field-emission microscope about 12–24 h later.

One immediate and recurring observation was a very smooth deposit appearance directly after deposition but additional features later being observable in high-resolution imaging.
Figure S5c,d shows close-ups taken in the deposition SEM. Even though the resolution is limited here, our assumption is that the filament like structures visible in Figure S5e,f evolved only later from uncovered silver crystals at the deposit surface. This assumption is supported by the fact, that such features were not obtained for very long deposition times where silver migration to the interface was dominant. In case of remaining incompletely dissociated precursor also the in situ imaging itself could have an effect as this is equivalent to (very short) low-electron flux deposition. To rule out this possibility as much as possible, the deposition experiments were run during night. The GIS heating

was switched off in the early morning, the stage heating one hour later, and the in situ images were then only taken once the base pressure was recovered. Still, Figure S5f shows a sign of this imaging by the vertical line of silver crystals that corresponds to the image frame of Figure S5d. The reason here could be remaining precursor molecules adsorbed (chemisorbed) at the surface, as stated in the main manuscript, that are incompletely dissociated.
The evolution of filaments was also present for the case of the 5 min spot deposits. Such spot deposits are our paradigm test and focusing feature and depicted in an in situ close-up in Figure S5h. Figure S5i in high-resolution imaging shows the complete reorganization of the deposit by forming filament-like structures with the longest filaments arising from the deposit center (supposedly the largest silver reservoir) but also short filaments arising from the halo region. We cannot speculate on the specific reaction pathways nor even the actual point in time at which these reactions took place. We can only state that this must have happened sometime after the actual deposition process and before the high-resolution imaging. Hence, we leave this observation to further investigations. Finally, Figure S5j shows the halo region of the studied square deposit with an enhanced contrast. Here, it becomes apparent that also later electron-impact induces slight modification which provides another hint towards incomplete dissociation.

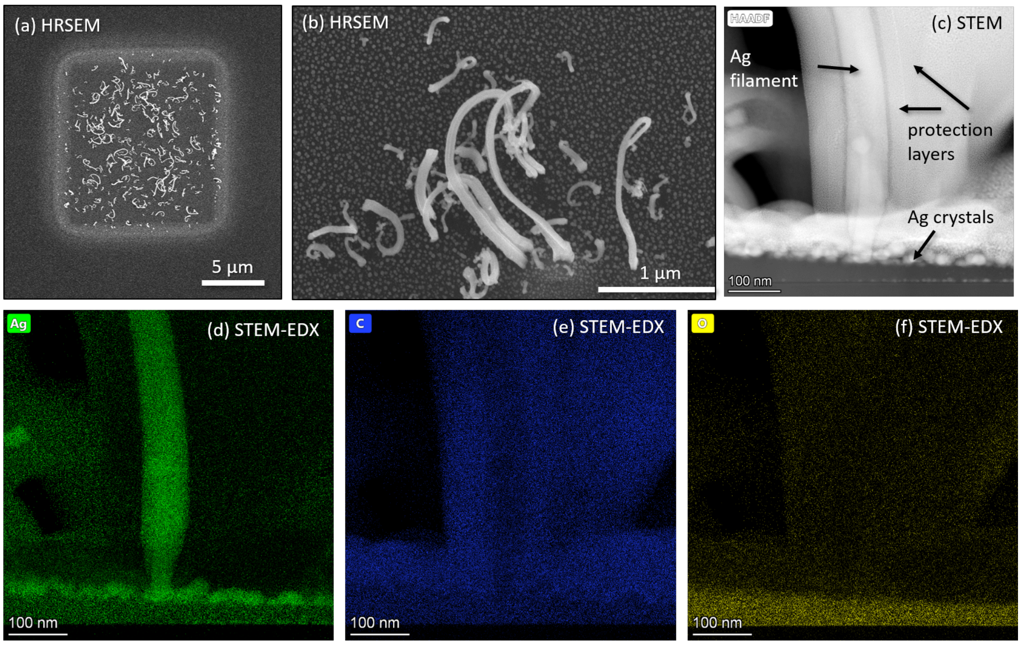

Figure S6: Morphology evolution. (a) and (b) HRSEM images of filaments that become visible at later inspection but were not present directly after deposition. (c) STEM HHADF image of a lamella prepared through one filament along with the corresponding EDX maps (d–f), which prove that the filament is silver.

The obtained filaments were further studied using STEM imaging and EDX spectroscopy. Here, the preparation of electron-transparent lamellas was extremely challenging. Still, one filament could be isolated and prepared and STEM-EDX could prove for the presence of silver. Interestingly, the bottom crystal from which the filament grew shows a smaller diameter than the filament itself. The results support the assumption that the filaments are from silver grown from particles present at the deposit surface but after the actual deposition process. This could rely on reorganization of

silver clusters due to their high mobility but also on thermally driven reactions of the incompletely dissociated precursor.

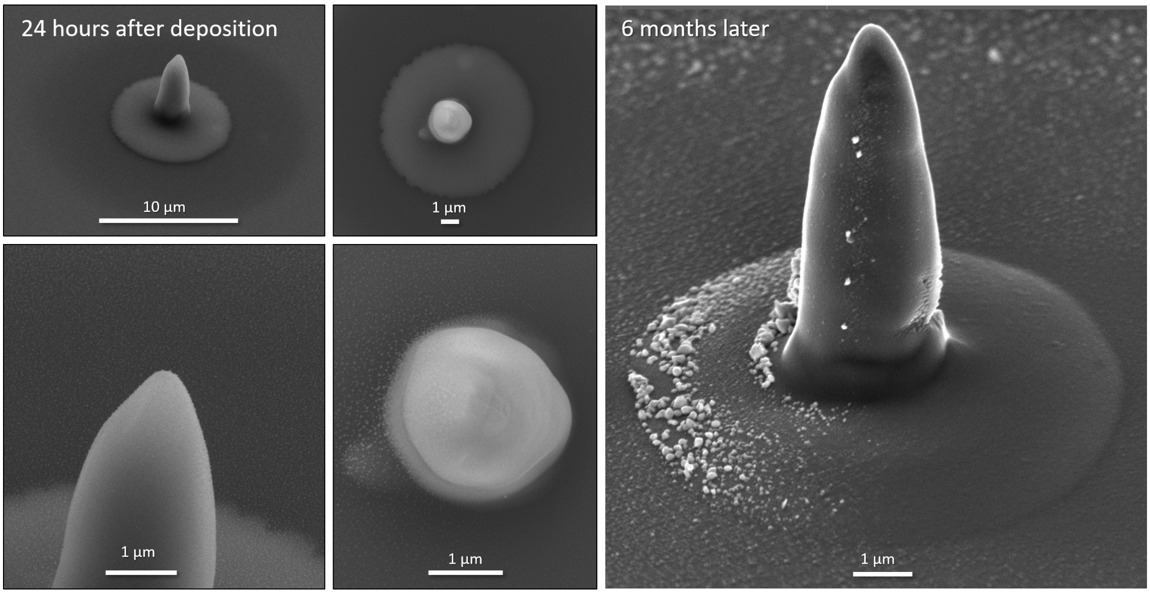

Figure S7: High resolution scanning electron micrographs of the 60 min spot deposit taken 24 h after deposition. The sample was taken out of the deposition SEM and imaged in the field-emission SEM. On the right side this is compared to a HRSEM image taken directly before the preparation of the cross section 6 months later.

The assumption that the mobility of silver and its reorganization plays an important role can be further supported by the time evolution of the 60 min spot deposit. Here, the first HRSEM images were taken about 24 h after the actual deposition process. Figure S7 depicts the spot deposit with close-up in top and tilted view. In the direction where the GIS was located, tiny particles are visible in halo region 1 on the left side of the deposit. (The angle of rotation is the same in all of these images.) On the top surface of the actual deposit even smaller particles are visible. After the surprising TEM results of the square deposits, a cross section was prepared for this sample as well, 6 months after the actual deposition process and the imaging. The HRSEM image taken directly before the preparation is shown on the right hand side of Figure S7 and depicts a significant growth of the particles. This growth was not an immediate result of imaging, which was also observed (cf. Figure S5j) but in that case the influence was minor compared to the morphological changes obtained here. Again, we cannot clarify based on the available data the exact reaction mechanisms here.
